# Is COVID-19 pushing us to the Fifth Industrial Revolution (Society 5.0)?

**DOI:** 10.12669/pjms.37.2.3387

**Published:** 2021

**Authors:** Zouina Sarfraz, Azza Sarfraz, Hamza Mohammad Iftikar, Ramsha Akhund

**Affiliations:** 1 Zouina Sarfraz, Fatima Jinnah Medical University, Lahore, Pakistan; 2 Azza Sarfraz, Department of Pediatrics and Child Health, Aga Khan University, Karachi Pakistan; 3 Hamza Mohammad Iftikar, Medical College, Aga Khan University, Karachi Pakistan; 4 Ramsha Akhund, Medical College, Aga Khan University, Karachi Pakistan

**Keywords:** Society 5.0, COVID-19, Development, Industrial, Revolution

## Abstract

The coronavirus disease 2019 (COVID-19) pandemic may further promote the development of Industry 4.0 leading to the fifth industrial revolution (Society 5.0). Industry 4.0 technology such as Big Data (BD) and Artificial Intelligence (AI) may lead to a personalized system of healthcare in Pakistan. The final bridge between humans and machines is Society 5.0, also known as the super-smart society that employs AI in healthcare manufacturing and logistics. In this communication, we review various Industry 4.0 and Society 5.0 technologies including robotics and AI being inspected to control the rate of transmission of COVID-19 globally. We demonstrate the applicability of advanced information technologies including AI, BD, and Information of Technology (IoT) to healthcare. Lastly, we discuss the evolution of Industry 4.0 to Society 5.0 given the impact of the COVID-19 pandemic in accordance with the technological strategies being considered and employed.

The role and impact of Industry 4.0 is growing as the world enters different phases of the coronavirus disease 2019 (COVID-19) pandemic. Every business, including the healthcare sector, is disrupted which is reflected by the dwindling global operations and dearth of Smart Manufacturing (SM) technologies.^1^ Major industrial shifts throughout history were driven by natural disasters or outbreaks of infectious diseases threatening public health security.^2^ The evolution of humankind is dominated by industrial revolutions, one after the other, which have changed the face of the modern world. The fifth industrial revolution is dawning upon the world in unforeseeable ways as we increasingly rely on Industry 4.0 technologies including Artificial Intelligence (AI), Big Data (BD), the Internet of Things (IoT), digital platforms, augmented and virtual reality, and 3D printing.^3^ Society 5.0, known as the super-smart society, may be the final bridge between machine and man.^4^ Among the technologies of Society 5.0, the most important are AI, robotics, 3D printing and digital platforms. BD and AI have already changed the face of white and blue-collar jobs.^5^ The current generation is gearing itself towards a more personalized future due to the recent adoption of AI algorithms.

The first industrial revolution began in 1780 with the introduction of mechanical production facilities using steam and water power.^6^ In 1870, the second revolution led to mass production of electricity to support the division of labor. The technological advances across all the industries included the use of new materials, and energy sources such as coal, steam engine, petroleum, and electricity. The revolution was marked by efficient and mechanized production requiring smaller expenditure of human energy. The third industrial revolution commenced in 1970 when the first Programmable Logistic Controller (PLC) Modicon 084 was built.^6^ It was marked by the automation in production of Information Technology (IT) and electronic systems. The fourth industrial revolution led to the generation of Cyber-Physical Systems (CPS) and the development of technologies such as BD, IoT, 3D printing and AI. CPS, an Industry 4.0 tool, is an automated, and connected device capable of learning from the physical environment which is responsive and can act independently. The IoT is a network over which the CPS connects to the internet in an auditable and secure manner. Finally, a Smart factory uses a combination of cyber physical systems and humans with support from intelligence and automation. The relevance of Industry 4.0 is investigated because smart Society 5.0 is built upon Industry 4.0, forming a community that is human-machine centered.^4^ Fig.1 depicts the timeline of industrial revolutions and the emergence of society 5.0.

**Fig.1:**
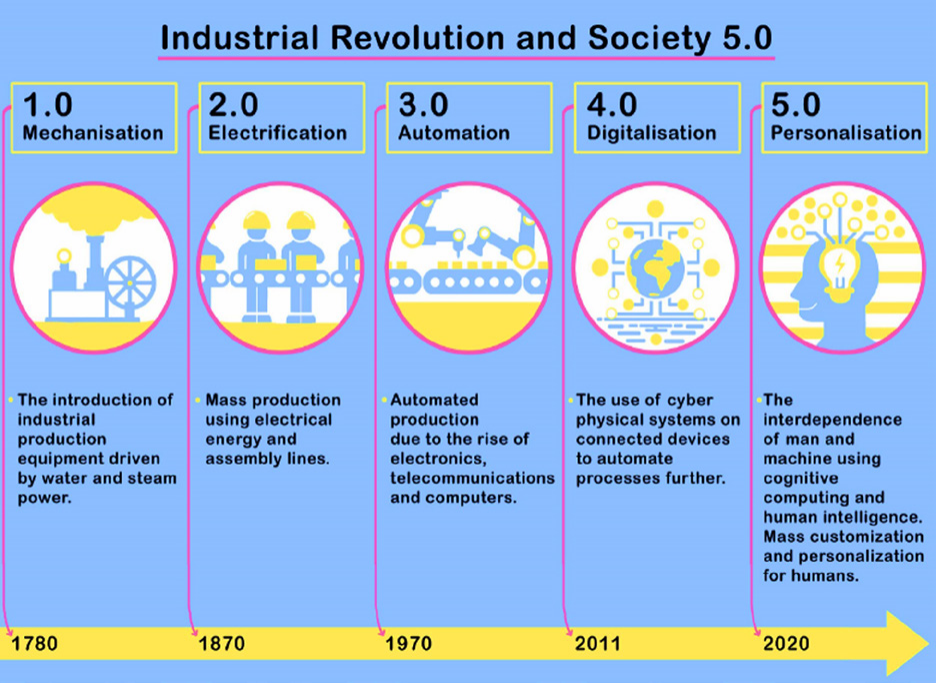
The timeline of industrial revolutions and the emergence of society 5.0 amid the COVID-19 pandemic in 2020.

Since its introduction in 2011, Industry 4.0 has led to rediscovered growth and transformation in technology. The core emphasis enabling Industry 4.0 sustainability and development is the exchange of information between companies, machines and humans. Production companies and the health industry have experienced the digitization threshold.^7^ The person-centered application of Industry 4.0 to digital health and care services has gained traction through an integrated care paradigm built on technological capabilities.^8^ Recent development and deployment of Point of Care (POC) diagnostics for screening of the COVID-19 outbreak may help alleviate the existent stress on the health system.^8^ Emerging health innovations such as AI technology coupled with POC diagnostics can enable and support self-testing in isolation post-exposure.^8^ Virtually, all objects today are equipped with sensors that identify the location or status of an individual at any time.^9^ Information is being processed and made available in larger volumes than it was in the twentieth century. The COVID-19 pandemic may steer the world into the next industrial revolution creating Society 5.0 where the amount of information may be impossible for processing by Industry 4.0 technologies. AI holds strong relevance as its computing power has notably doubled every two years.^9^ AI has not only become “smart enough” to win humans at games, but extracts meaning out of BD. The implications that smart technology and ongoing transformation in traditional manufacturing have on COVID-19 and Society 5.0 are worth identifying.

Medical diagnostics and AI are well-designed systems being used in mobile technology for contact tracing and data collection technologies. Current digital tools have shown promising results in managing infectious outbreaks. However, improved human application via society 5.0 can improve coping with the impact, speed, and scope of infectious disease outbreaks. While social distancing remains the most useful method to reduce the rate of transmission of COVID-19, the existing Industry 4.0 technology has shown great potential to control the pandemic. To maximize the sustenance of Industry 4.0 and its application, China’s AI-based thermal imaging cameras can successfully scan public places and identify potentially infected individuals.^10^ AI-based computer vision cameras are being employed to calculate whether people are socially distancing in public areas. Specific technologies that show promise to cope with the pandemic include AI and mobile technology products. Partners Healthcare is an AI-based screener app for COVID-19 that screens potential patients that may require further evaluation.^11^ The Canadian AI model BlueDot presented an early warning on 31 December 2019 that the coronavirus disease (2019) would spread globally.^12^ A proposal to curb the impacts of the coronavirus includes data collection tools to aid in predicting outbreaks, promoting contact tracing, symptom checking, and establishing vulnerabilities.

With the onset of society 5.0, robots and drones may deliver medical supplies to health facilities, meals, and medicine to infected patients.^4^ Industry 4.0 and the technological revolution addresses certain problems in Pakistan’s healthcare system such as dwindling levels of patient care or lack of resources. Nationally, Industry 4.0 technology ensures every vital step to curb health impacts to limit human exposure. The international application of cryptocurrencies such as Bitcoin are successful alternatives for the financial sector in emerging economies such as Pakistan. Similarly, mobile money can allow vendors and customers to go cashless. The use of AI and blockchain in healthcare is evident in the following areas: health data analytics, remote patient monitoring, education, biomedical research, management of electronic medical records, drugs and pharmaceutical supply chain management.^13^ Nanotechnology, the secret to the fifth industrial revolution and the future of the next generation, and advanced materials play a crucial role in therapeutics, rapid diagnostics, surveillance and monitoring, developing novel forms of PPE, and vaccines.^14^ Recently, an Italian company named Nanotech Surface produced a nanomaterial-based sanitizer that kills bacteria for months after it is applied to any surface. Similarly, the Czech Respilon Group designed respiratory masks that were capable of killing viruses rather than just trapping them. Such events suggest that Industry 4.0 has already taken center stage across the world, thus plunging the world into Society 5.0.

Reports from Japan suggest that by 2050, 40% of its population will be 65 years and older.^15^ The super-aged society will have challenges as pharmaceutical companies may have inadequate resources and supplies to address the demands of the older generation. The solution may be Society 5.0’s smart robotics such as assist systems, which may help prolong independent life, and robotics that can take over elderly care entirely. AI can be the setting stone in developing drugs to support the elderly. Japan considers that the development of Society 5.0 has begun.^16^ China has considerably enhanced its efforts of executing a balanced strategy by using the tools of tomorrow. The country has harnessed the power of robotics, which have been instrumental in reducing interpersonal communication during pandemics. China has exemplified the latest phase of human evolution by using robotics to deliver food, medication, and incorporate BD. The incorporation of such technology has enabled China to create resourced databases for predicting infectious risks along with incorporating extensive screening protocols.^17^

The literature pertaining to the capability of Industry 4.0 capabilities and its extension to Society 5.0 in health settings is limited. While connecting the health system using Society 5.0 tools such as AI and robotics may be beneficial, a large part of the world including developing countries such as Pakistan do not have potential access to such innovations. Developed countries have an internet penetration rate of 81%, whereas only 17.5% of the population has access to the internet in developing countries.^18^ Given these trends, it is essential that the concept of new healthcare and manufacturing systems does not decrease access to basic services at the primary, secondary or tertiary level of care in Pakistan. On the one hand, technological inventions seem feasible in applicability. On the other hand, the lack of funding, adequate managerial measures in healthcare sectors, and skilled technological support are potential limitations for the successful application of Society 5.0 technological tools in Pakistan.

Society 5.0 has already taken the stage amid the COVID-19 pandemic. The concept of a super-smart society rests on advanced IT technologies including AI, IoT and robots that will be utilized by manufacturers, healthcare sectors, and other sectors. The availability of such vast data can transform society into a human-centric one. The fifth step in the evolution of humankind may commence in the midst of a global health crisis.^19^ The world was once a hunting society, and settled down as an agricultural one. As the communities bridged towards an industrial society, mass production was given precedence. Today, the majority of the world is dependent on an information-centric society. However, the creation of knowledge is still controlled by humans. The world is on the verge of breaking through the super-smart fifth society. COVID-19 and the incorporation of Society 5.0 may bring positive impact upon development measures in communities. The unforeseen benefit of the COVID-19 pandemic is the ability to experiment with cooperative approaches to technology across borders that leads to a more sustainable, safer, and inclusive global future.

## Author`s Contribution:

The authors contributed equally to all aspects of the article.
